# Recent Advances in Microfluidics for the Early Detection of Plant Diseases in Vegetables, Fruits, and Grains Caused by Bacteria, Fungi, and Viruses

**DOI:** 10.1021/acs.jafc.4c00454

**Published:** 2024-06-14

**Authors:** Xiaohan Zhao, Lingzi Zhai, Jingwen Chen, Yongzhi Zhou, Jiuhe Gao, Wenxiao Xu, Xiaowei Li, Kaixu Liu, Tian Zhong, Ying Xiao, Xi Yu

**Affiliations:** † State Key Laboratory of Quality Research in Chinese Medicine, Macau University of Science and Technology, Taipa, Macao 999078, People’s Republic of China; ‡ Faculty of Medicine, Macau University of Science and Technology, Avenida Wai Long, Taipa, Macau 999078, People’s Republic of China; § Department of Food Science & Technology, National University of Singapore, Science Drive 2, Singapore 117542, Singapore; ∥ Wageningen University & Research, Wageningen 6708 WG, The Netherlands

**Keywords:** lab-on-a-chip, point-of-care, agri-food safety, detection methods, plant disease

## Abstract

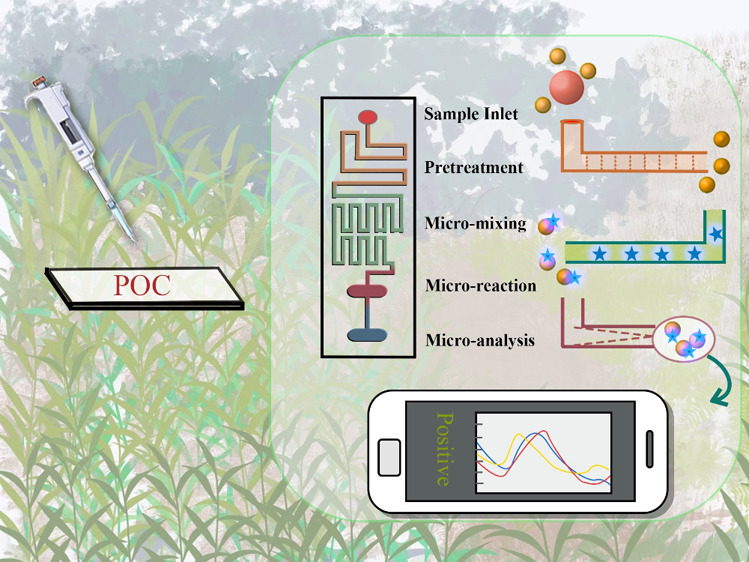

In the context of global population growth expected in the future, enhancing the agri-food yield is crucial. Plant diseases significantly impact crop production and food security. Modern microfluidics offers a compact and convenient approach for detecting these defects. Although this field is still in its infancy and few comprehensive reviews have explored this topic, practical research has great potential. This paper reviews the principles, materials, and applications of microfluidic technology for detecting plant diseases caused by various pathogens. Its performance in realizing the separation, enrichment, and detection of different pathogens is discussed in depth to shed light on its prospects. With its versatile design, microfluidics has been developed for rapid, sensitive, and low-cost monitoring of plant diseases. Incorporating modules for separation, preconcentration, amplification, and detection enables the early detection of trace amounts of pathogens, enhancing crop security. Coupling with imaging systems, smart and digital devices are increasingly being reported as advanced solutions.

## 1 Introduction

The global population is expected to exceed 9.7 billion by 2050, increasing the demand for food quantity and quality. According to statistics from the Food and Agriculture Organization (FAO), there is a need for a 60–110% increase in agri-food production to satisfy demand.^1^ Despite challenges such as climate change, land depletion, and environmental pollution, plant diseases continue to pose threats, impacting food security and contributing to economic losses. The FAO estimates that major crops (rice, wheat, maize, potatoes, and soybeans) suffer an annual loss of 13–22% due to various plant diseases, resulting in economic losses of approximately $220 billion and affecting more than 800 million people globally.^2^ Human activities exacerbate this crisis, spreading pathogens globally. Modern monoculture practices, while reducing production costs, also promote the occurrence of infectious plant diseases.^3^ Bacterial, fungal, and viral infections have increased in recent years, causing major diseases such as downy mildew, blast disease, and citrus greening disease (as shown in Table 1).^4−13^ Swift detection and control are vital for ensuring agricultural safety, food security, and public health.

**Table 1:** List of Attributes of Different Plant Diseases Caused by Pathogens

crop	disease	pathogen	type of pathogen	symptoms
lettuce	downy mildew	*Bremia lactucae*	fungus	light green to yellow angular spots appear on the surface of the leaves, with white fluffy growth of pathogens on the underside
potato	late blight	*Phytophthora infestans*		the edges of the lower leaves appear as watery spots and gradually expand
potato	brown rot disease	*R. solanacearum*	bacteria	appear as blossom blight or shoot dieback
wheat	rust diseases, stripe rust, powdery mildew	*Puccinia triticina*, *P. striiformis*, *Blumeria graminis*		small brown pustules develop on the leaf blades in a random scatter distribution, a mass of yellow to orange urediniospores erupting from pustules arranged on leaves, white patches of fungal growth and purple to reddish blotches develop on the leaf Leaf edges curl upward, exposing the white, powdery fungal growth
rice	blast disease	*Pyricularia oryzae*		the lesion with brown border is enlarged and the stem is broken
tomato	damping off	*P. aphanidermatum* (Edson) Fitz	fungus	groups of seedlings may die in roughly circular patches, the seedlings sometimes having stem lesions at ground level
winter wheat	stem rust	*Puccinia graminis* f. sp. *tritici* (Pgt)	phylum Basidiomycota within the kingdom Fungi	the spores on the leaf surfaces that range from orange to dark-red in color
grapevines	grapevine leafroll disease	grapevine leafroll-associated virus 3 (GLRaV-3)		leaves show red and reddish-purple diskolorations, fruit ripening delayed and quality decreased
cotton	cotton root rot	*Phymatotrichum omnivorum*		the plant suddenly wilts, all leaves droop and die within a day or two
citrus	Liberibacter Infection (citrus greening disease)	*CandidatusLiberibacter asiaticus*		size reduction, pale yellowing, mottled or variegated greenless erect small leaves, followed by leaf drop and later stages of twig death
pear	*Alternaria* rot disease	*A. alternata*		*Alternaria* rot

Traditional plant disease control relies on visual assessments to identify pathogens and disease hallmarks, which are inefficient and demand specialized expertise.^14^ In recent years, there has been a significant increase in the advancement of modern instrumental analysis methods.^15−19,19^ These techniques rely heavily on large-scale benchtop equipment, which is difficult to use for on-site tests. Smaller spectrum techniques such as chemiluminescence, colorimetry, and fluorescence are portable but may have no separation module and compromise accuracy.

Microfluidic chips, also known as microfluidics, miniaturized total analysis system (mTAS), or lab-on-a-chip (LOC) chips, have revolutionized sample separation and detection.^20^ These “chip laboratories”, comprising microchannels, microvalves, micropumps, and detection units, enable high sensitivity, high throughput, and rapid detection. Microchannels, fundamental to these chips, are the main structures that can be realized on a small device scale. Their shape and size can be designed as needed to construct compact structures that allow multiple trials to be run on a single chip, thereby increasing throughput. Moreover, microchannels contribute to reducing the consumption of biological samples and reagents required for detection, thus decreasing costs. In addition, the reduction in the channel length is associated with faster analysis and the corresponding time. Microvalves are usually driven by electric, capillary, or pneumatic methods. These materials can switch microchannels as needed to achieve highly precise quantitative control and separation of samples.^21^ The detection unit (sensor) captures and analyzes target molecules or cells in samples through structures such as microarrays or micropores. Based on this intelligent structure, microfluidic technology can often improve the application deficiencies of traditional detection techniques, such as long time consumption, complex operation, and susceptibility to contamination, providing quick and convenient plant disease analysis. The use of microelectromechanical systems (MEMS) originating from integrated circuit manufacturing has facilitated biochemical analysis miniaturization, automation, and integration.^22^ The concept of the perfect combination of LOC and point-of-care testing (POCT) was based on the unique fluid phenomena in microfluidics. By incorporating a sample inlet, pretreatment, micromixing, microreaction, and microanalysis into a single chip, the microfluidic platform can detect plant diseases in real time at once. This makes real-time infection information available to nonexperts and enhances the productivity of plant disease detection. The development of various sensor systems lays the groundwork for the early detection and diagnosis of plant diseases.^14^

Given the promising potential of LOC for addressing agri-food pathogen screening and enhancing food security, this review offers an in-depth exploration of recent advancements in this field. It covers microfluidic device principles, methods, and mechanisms in plant disease detection, explaining the characteristics, strengths, and limitations of different fabrication materials.

Research progress in detecting microbial diseases causing plant diseases based on LOC, categorized by the type of LOC, is then discussed. This review concludes by presenting prospects for this rapidly developing field, emphasizing current challenges. It is believed that this comprehensive review will bridge gaps among materials science, microfluidics research, and practical needs in agricultural and food security, facilitating the development and practical implementation of portable and POCT devices in modern agriculture.

## 2 Methods

### 2.1 Literature Search Strategy and Eligibility Criteria

The purpose of the literature search was to identify all the studies describing microfluidic devices applied to agri-food disease detection from the last ten years. The literature search was carried out by consulting the PubMed and Web of Science databases. To maximize coverage of the relevant literature, we used the following search terms: (lab-on-a-chip OR microfluidic OR “miniaturized total analysis system” OR chip OR loc) AND (“plant disease” OR “crop disease” OR “agricultural disease” OR “agricultural food disease” OR “plant pathogens” OR “plant bacteria” OR “plant fungus” OR “plant virus”).

Inclusion criteria were defined to select the studies. Specifically, we included studies from the past decade (2013–2023) describing the use of microfluidics and studying diseases of agricultural products (fruits, vegetables, and grains). The inclusion criteria for the study were as follows: (a) research involving the application of microfluidics for agricultural disease detection and (b) published between 2013 and 2023. The exclusion criteria were as follows: (a) not primary or peer-reviewed scholarly articles, (b) review articles, and () research content inconsistency.

### 2.2 Search Results

**Figure 1:**
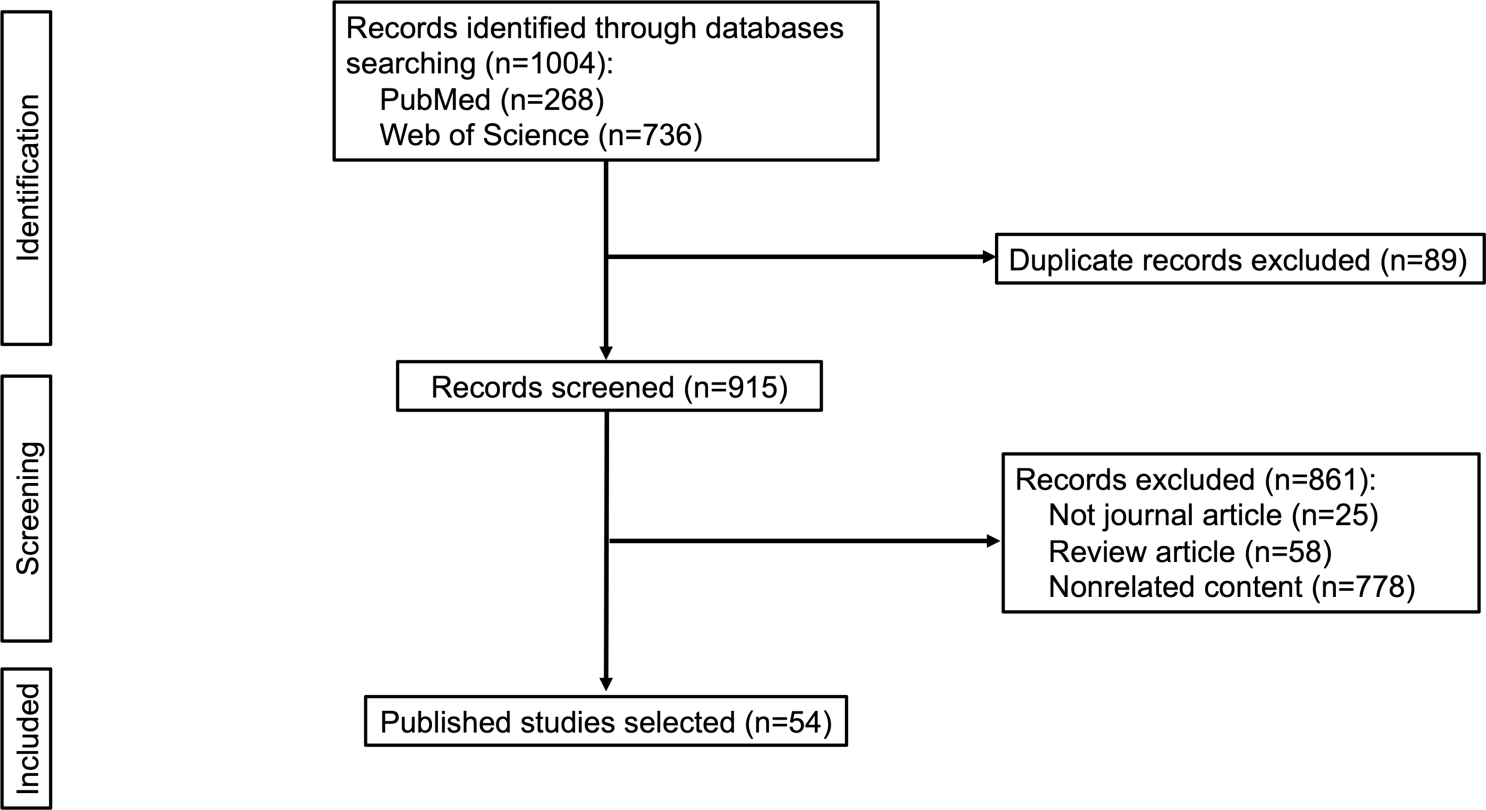
Flowchart of the study selection.

According to the literature search strategy, a total of 1004 studies were found (268 in PubMed and 736 in the Web of Science). Among them, 89 articles were excluded because they were duplicates. After the full texts were reviewed and the eligible articles were screened further, studies that met the inclusion criteria were selected. Of the remaining 915 records, 25 records were excluded because they were not primary or peer-reviewed scholarly articles, and 58 review papers were excluded. After full-text reading, 778 articles were excluded because their contents were related to plant physiology, plant genomics, metabolomics, the detection of foodborne pathogens, reports of plant diseases, etc., which are not relevant to our topic. In total, we included 54 studies that met our eligibility criteria for analysis (Figure 1).

## 3 Mechanism for Using Microfluidics to Detect Plant Diseases

### 3.1 Definition and Classification of Microfluidics

Microfluidics refers to the scientific discipline involving the manipulation of fluids within systems of micrometer dimensions.^23^ Microfluidics can be broadly divided into three categories: (i) continuous flow, (ii) droplet-based, and (iii) digital microfluidics.^24^ Continuous microfluidic devices consist of permanently etched microchannels and peripheral devices (such as micropumps and microvalves) used to manipulate the fluid flow within these devices. Droplet-based microfluidic systems rely on the generation of droplets in microchannels by using two or more immiscible fluids at T-junctions. However, digital microfluidic systems are fundamentally different because they provide the movement and control of discrete droplets on a planar array of electrostatically driven electrodes. The development of microfluidic chips also benefits from the advancement of materials science.^25^

**Figure 2:**
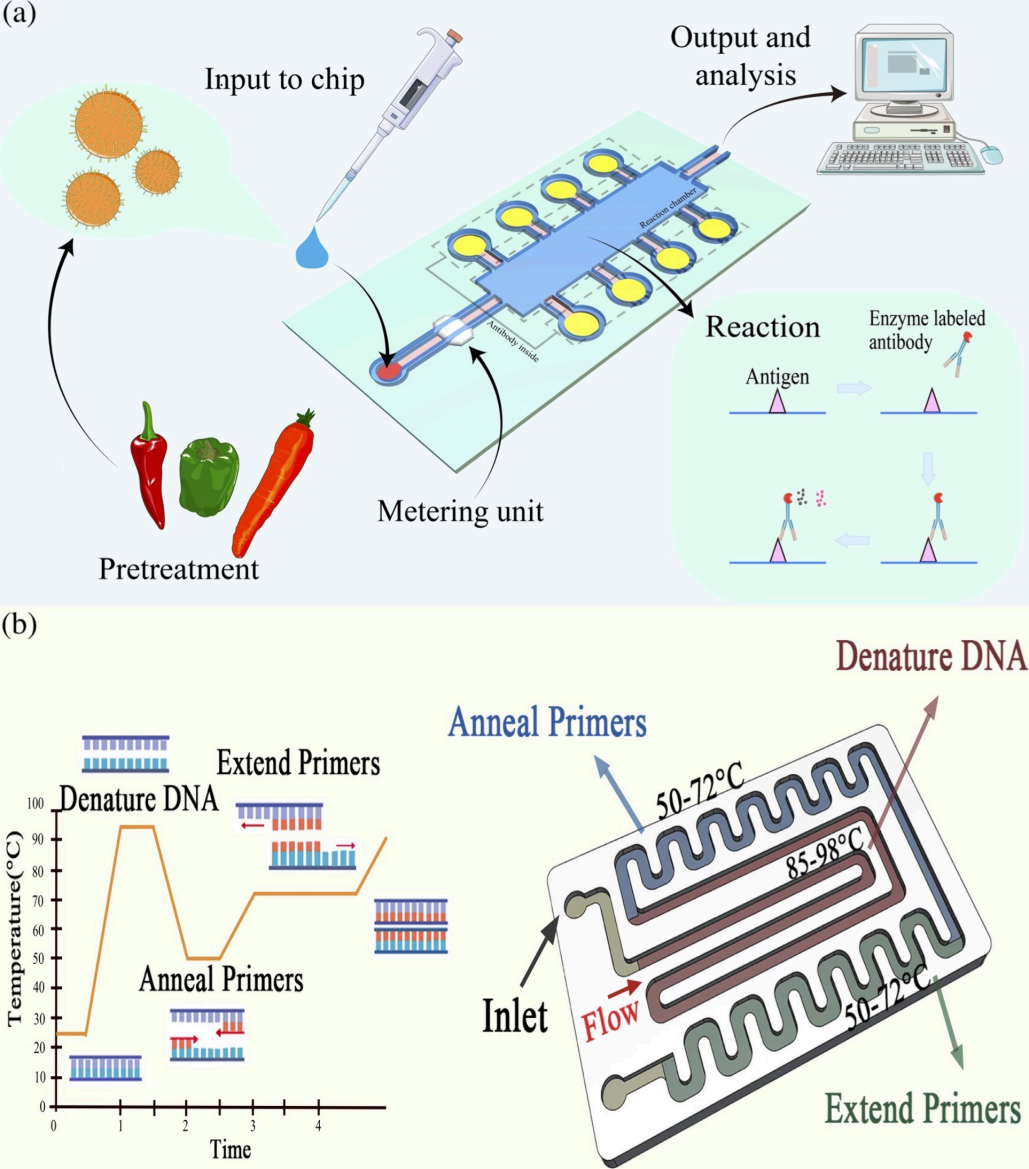
(a) Enzyme-linked immunosorbent assay (ELISA) and (b) Polymerase chain reaction (PCR) revealed LOC.

Microfluidics offers a unique platform for an enzyme-linked immunosorbent assay (ELISA) and polymerase chain reaction (PCR) through precise control of fluid flow and sample handling in tiny channels and chambers (as shown in Figure 2). Compared with PCR, the integration of ELISA with microfluidics has different steps and implementation methods. Initially, samples enter the microfluidic chip and undergo preprocessing steps, such as mixing, dilution, or incubation to prepare for ELISA analysis, whereas PCR typically entails mixing, heating, or stirring. Microfluidics ensures the precise addition and mixing of reagents, antibodies, substrates, and primers, enhancing accuracy and stability. The channels and chambers are designed to sequentially execute various steps of ELISA, such as sample loading, antibody–antigen binding, washing, and the substrate reaction. In contrast, PCR typically requires thermal cycling to amplify DNA at different temperatures. In summary, microfluidics provides flexible and precise solutions for diverse analyses.

The mechanism for using different microfluidics to detect plant disease is detailed in the following sections.

### 3.2 Immunological Methods of Integrated Microfluidics for Screening Plant Diseases

The use and development of immunological methods have attracted great interest due to their high selectivity and sensitivity. ELISA is the most commonly used method among various immunoassays and is typically performed in a 96-well microtiter plate. ELISA detects pathogens via immune and enzymatic reactions and assesses infection levels through colorimetric responses. Initially, applied for viral identification, ELISA has been expanded to analyze plant diseases. Figure 2a shows the general application of ELISA to LOC. Researchers are striving to improve the limitations, cross-reactivity, and sensitivity of these antibodies. Microfluidics aims to expedite and miniaturize ELISA tests.^26^ ELISA-integrated microfluidics shows promise for plant disease assessment, offering high-sensitivity analysis through enhanced reaction efficiency, simplified procedures, and a shortened analysis time. Challenges remain, requiring improvements in sample/reagent efficiency, sensitivity, and integration with user-friendly platforms.

A microfluidic sandwich ELISA for watermelon virus detection offered 2–12.5 times greater sensitivity, suggesting automation through adaptable robotic liquid handling systems.^27^ In 2018, micro/nanofluid single-molecule ELISA demonstrated precise detection (∼100% capture) of specific individual molecules (proteins), representing a significant innovation in analytical chemistry.^28^ Subsequent advancements include acoustic streaming-based microfluidics, which exhibit potential for multiplexing and automatic immunoassays.^29^ Most recently, smartphone-enabled colorimetric ELISA (c-ELISA) presented a fully automated sensing process and user-friendly application.^30^

### 3.3 Nucleic Acid Testing-Based Microfluidics for Analysis of Pathogens in Agricultural Plants

PCR amplification of the nucleic acid sequences of pathogens is the most widely used molecular technology and has specificity, universality, and effectiveness in the analysis of pathogens. Conducting nucleic acid processing via microfluidics reduces contamination risks and thermal cycle times.^31^Figure 2b shows the steps of PCR to achieve amplification of DNA synthesis in vitro through multiple cycles of denaturation, annealing, and extension and a general explanation of how to combine PCR and LOC. Other laboratory methods, such as loop-mediated isothermal amplification and quantitative polymerase chain reaction (qPCR), have also been explored.^26,32,33^ Challenges in PCR-based microfluidics include user interface simplicity and the potential for integration with DNA arrays or sequencing methods on the same platform.

Recent developments include a microfluidic biodevice system for the continuous identification of DNA targets. Researchers regenerated oligonucleotide arrays, enabling multiplexed detection with denaturation and laser-induced fluorescence. This fast microfluidic biodevice system allowed continuous analysis of gray mold and gummy stem blight.^34^ One study described fully automated and colorimetric bacterial detection on an integrated centrifugal microfluidic device.^35^ All molecular diagnostic processes, including DNA extraction and purification, DNA amplification, and amplicon detection, are integrated into a single disk. Silica microbeads embedded in disks can be used to extract and purify bacterial genomic DNA from contaminated samples, and loop-mediated isothermal amplification can be performed^32^ to amplify the specific genes of interest. The entire process was carried out automatically by using a disk laboratory and a small rotating instrument equipped with three heating blocks. Another study reported an integrated, self-driven microfluidic chip for digital LAMP.^36^ A small amount of template DNA and reagents is encapsulated in droplets or micropores, allowing the analysis of nucleic acid samples in a shorter time. The entire quantification process is automatically executed on this chip by a capillary force. The micropores consist of a poly(dimethylsiloxane) (PDMS) surface coated with a hydrophilic film, eliminating the need for external pumps. Additionally, the digital droplets are separated from each other through normally closed microvalves. This is the first reported rapid (30 min) and simple method for creating a hydrophilic PDMS surface, allowing digital LAMP to be performed on a self-driven microfluidic device. The detection limit was only 11 copies. Another study presented a rapid nucleic acid analysis microfluidic system based on real-time convective PCR.^37^ A hand-held real-time CPCR device was developed for nucleic acid amplification and real-time detection. The integrated microfluidic chip consists of reagent prestorage chambers, lysis washing chambers, elution chambers, and waste chambers. Magnetic bead-based nucleic acid extraction can be performed automatically on large test samples within a limited time. To expand the detection throughput, multiple hand-held real-time CPCR devices can be combined through a universal control system.

The CRISPR–Cas system possesses unique crRNA-guided sequence binding properties.^38^ CRISPR–Cas is one of the most commonly used biological recognition mechanisms, as it binds to dsDNA without the need for cumbersome denaturation, rehybridization, or annealing steps, making it highly suitable for specific nucleic acid recognition in plant disease detection. A novel platform that integrates droplet microfluidics with recombinase aided amplification (RAA)-assisted one-pot clustered short palindromic repeats has been introduced, together with the CRISPR-associated protein 13a (CRISPR/Cas13a) assay and a droplet encoding strategy.^39^ Taking advantage of CRISPR/Cas13a signal amplification and droplet confinement, the platform demonstrated high accuracy and sensitivity in simultaneously detecting nucleic acids for seven different types of foodborne pathogens. Meanwhile, by variation of the color of droplets, the number of bacteria detected at the same time is greatly improved. The researcher also claimed that considering advantages in high sensitivity, outstanding selectivity, and large-scale multiplexing, the CRISPR/Cas13a-based droplet microfluidic system could also be expanded and universalized for identifying other bacteria in other fields, which is prospective for plant disease detection. The integration of CRISPR detection with microfluidic systems to automate all liquid handling steps provides a solution to the problem of CRISPR detection relying on multiple operational steps such as nucleic acid extraction, amplification, and signal readout. However, there is currently a lack of fully integrated and validated detection platforms that are excellent candidates for CRISPR detection suitable for POCT.

### 3.4 Morphological Detection Based on Microfluidic Technology

Traditional morphological examination methods mainly include direct microscopic examination and staining. Direct microscopic examination involves taking samples from humans (or animals), making unstained sections, and observing them directly under a microscope. For fungi that are difficult to distinguish under a microscope, a staining examination is needed. Common fungal staining methods include Gram staining, Giemsa staining, PAS staining, etc. Due to the limitations of observation techniques, traditional fungal morphological detection has a very limited role in the detection and identification of fungi. Although morphological studies based on microfluidics play a crucial role in fungal plant disease identification and control, their scope is relatively limited. Additionally, it cannot handle high-throughput data and requires further integration with other technological approaches.

Currently, the rapid development of microscopic imaging technology and microfluidics has made it possible to observe the germination and growth of fungi in real time. To explore the growth kinetics of single cells, a study proposed a microfluidic platform for capturing single sporangia and allowing single hyphae to grow in spatially separated channels.^40^ Another system produced highly monodisperse droplets for high-throughput screening of filamentous fungi based on enzyme activity.^41^ Delayed microscopy in a microfluidic chip allowed in-depth phenotypic analysis of microorganisms, aiding in studying the growth, germination, and spore formation of the fungus causing sudden death syndrome in soybean plants.^42^ Recently, there has been a report of fungal growth at the single hyphal scale in microfluidic devices.^43^ In microfluidic devices, nutrient and water supplies can be precisely controlled, and time-lapse microscopy allows simultaneous monitoring of the soil isolate *Talaromyces* helices and the growth of dozens of hyphae of model fungi through parallel microchannels. This research can inspire the study of key factors controlling fungal growth. Microfluidic systems have also been developed to study the growth and enzyme secretion of individual hyphae of filamentous fungi.^44^ The hyphae of filamentous fungi exhibit extensive branching, making it difficult to observe and analyze hyphae by controlling their growth. Microfluidic systems confine hyphae to individual channels for observation and study of the relationships among fungal growth, morphology, and enzyme productivity. This microfluidic system is capable of visualizing in real time the dynamics of hyphae and enzymes during carbon source exchange and the quantitative kinetics of gene expression and is applicable to many biological systems in agriculture. Furthermore, to address the limitations of high-resolution dynamic imaging of fungal–fungal interactions on agar surfaces and to obtain real-time experimental access to FFIs at the hyphal level, a multifunctional microfluidic platform has been developed to measure the hyphal interactions between *F. graminearum* and *R. solani* in real time.^45^ The microchannel geometry is utilized to enhance the visibility of hypha growth and to provide control channels to allow comparisons between local and systemic effects. Microscopy image analysis can be used to observe fungal interactions in real time. This study also demonstrated the multifunctionality of the device under dry and nutrient-deficient conditions, opening up new opportunities for studying the relationships between fungi.

### 3.5 Others

One study reported an integrated microfluidic device with organic photodiodes and organic light-emitting excitation sources for fluorescence-based detection of specific pathogens.^46^ Another novel microfluidic integrated filter for sensitive biological load detection using trypan blue oxidation–reduction reactions. This microfluidic device was manufactured using microwave-induced thermal-assisted bonding in a simple, low-cost, and fast manner.^47^ In addition, a wireless, standalone device and disposable microfluidics device based on electric and nonelectric microsphere detection enabled rapid parallel readings of selected virus variants.^48^ This system can economically, disposably, and simultaneously detect up to six different viruses, particles, or variants in a single test and collect data using commercially available Wi-Fi-enabled camera-integrated devices.

## 4 Materials Used in Different Microfluidics for Plant Disease Screening

In the application of microfluidic technology, the choice of chip material is very important. Materials with ideal properties not only are easy to manufacture and cost-effective and have short detection times but also maximize the functions of separation, enrichment, and detection of the target substance. This allows the chip to be better used as a diagnostic device or integrated unit for POCT.^53^

Inorganic materials were first used to make microchannels. This is due to the stable silica and glass surface properties and the reusability of the material. However, as shown in Table 2, these two materials have rarely been used in agri-food pathogen detection chips. One possible reason is that with the development of microfluidics manufacturing technology, silica and glass materials are no longer ideal materials for LOC manufacturing. For example, the high precision required by POCT technology often requires specific functional groups for functionalization, and these materials are not easily modified.^54^ Therefore, there are a variety of new materials with better processes, characteristics, and integration degrees to choose when chips are applied to detect plant disease pathogens. The polymer material is the material of choice for LOC applications due to its suitable properties, such as biocompatibility, environmental friendliness, and ability to be produced in large quantities. The most prominent of these PDMS materials were found by researchers to have beneficial properties such as high optical transparency, low toxicity, and permeability.^55^ In addition, paper-based microfluidic devices are also a focus of POCT, especially in resource-limited environments.^53^ Applications related to new materials such as hydrogels have received increased attention in recent years.^56^ With the manufacture, modification, and application of different materials, the chip will also have better performance.

**Table 2:** Summary of the Use of Metrofluidic Chips for the Detection of Agri-Food Pathogens^a^

	pathogen	analytes	agri-food/biomarker	chip materials	combined detection techniques	sample volume	detection time	LOD	LOQ	accuracy	specificity	capture efficiency	ref
fungus	*Botrytis cinerea*	organic acid (azelaic acid)	grapes	PDMS	colorimetric monitoring	25 μL	under 10 min	4.8–9.9 nM					(72)
	*Sclerotinia sclerotiorum*	Ascospores	canola crop	borosilicate glass	electrochemical impedance spectroscopy and coplanar microelectrodes	10 μL		single pores				87%	(81)
	*Botrytis cinerea*, Erysiphe necator	organic acid (salicylic, azelaic, and jasmonic acids)	grapes	PDMS	transmission measurements, fluorescence-based assay/nanoparticle conjugation, enzymatic assay, immunoassay	25 μL	under 7 min	4.4 nM–15 μM					(49)
	*Botrytis cinerea*	spores	tomato	PDMS	lens-free diffraction image processing	100 particles						70.65–100%	(74)
	*Botrytis cinerea*, *Pseudoperonospora cubensis*, *Podosphaera xanthii*	spores	tomato and cucumber			100 particles						75.7–89.4%	(76)
	mold and gray mold	spores	strawberry	PDMS								76–89%	(77)
	rice blast fungus	spores	rice	PDMS	lens-free diffraction image processing					94%			(75)
	*Magnaporthe grisea* fungus and *Ustilaginoidea virens* balls	spores	rice	PDMS	microscopic hyperspectral imaging technology	5 × 10^6^ spores mL^–1^, take 10 mL of the prepared suspension				96%	95%	82.67–80.70%	(78)
	rice false smut	spores	rice	PDMS		particle: 100 particles							(79)
	*Ustilaginoidea virens*, *Magnaporthe grisea*, and Aspergillus niger	spores	rice	PDMS	impedance detection	particle: 100 particles				97.78%	96.67%		(80)
	*Phytophthora cactorum*	*Phytophthora cactorum*	strawberry leaves	LFD	recombinase polymerase amplification		40 min	100 fg of genomic DNA					(88)
	*Ustilaginoidea virens*	spores	rice	LFD	recombinase polymerase amplification	particle: 100 particles		1 × 10^2^–1 × 10^5^ CFU mL^–1^			100%	98%	(50)
	rice smut spores	spores	rice	PDMS	loop-mediated isothermal amplification^32^	0.2 μL	72 min	100 copies/reaction			100%	78%	(89)
virus	*Citrus tristeza* virus (CTV)	capsid protein	citrus crops		magneto-immunoassay methodology	340 μL		0.3 fg mL^–1^					(61)
	*Citrus tristeza* virus (CTV)	viruses	citrus	polystyrene	electrochemical immunosensor based on the use of an antibody	20 μL	60 min	0.27 nM	0.97 n024110 M				(90)
	*Acidovorax citrulli* (Ac), watermelon silver mottle virus (WSMoV), and melon yellow spot virus (MYSV)		Watermelon		sandwich ELISA	100 μL	90–140 min	4 × 10^5^ CFU mL^–1^, 625 ng mL^–1^, and 80 ng mL^–1^	6 × 10^9^ WSMoV particles/mL and 8 × 10^8^ MYSV particles/mL				(27)
	banana bunchy top virus (BBTV)	viruses	banana	FLD				0.13aM					(52)
	*Ampelovirus* Grapevine leafroll-associated virus 3 (GLRaV-3), *Nepovirus* Grapevine fanleaf virus (GFLV)	viruses	grapevine plant	PDMS	impedance detection			1:50 dilutions for GFLV, 1:100 dilutions for GLRaV-3					(91)
bacteria	*Xanthomonas arboricola* (XA)	bacteria	walnut trees	PDMS	sandwich immunoassay and electrochemical		30 min	1.5 × 10^2^ CFU mL^–1^					(82)
	spore-forming bacteria	bacteria		PDMS and glass	laser tweezer Raman spectroscopy (LTRS)								(92)
	*Pectobacterium atrosepticum* (Pba)	bacteria	potato	PDMS	electrochemical impedance spectroscopy (EIS)			10^4^ CFU mL^–1^					(83)
	*Xylella fastidiosa* subsp. *pauca* strain CoDiRO	Bacteria	Olive tree	PDMS	Electrochemical impedance spectroscopy (EIS)	5 μL		1.3 × 10^3^ CFU mL^–1^					(84)

a Blank entries indicate information not provided.

### 4.1 PDMS Microfluidics for Plant Disease Screening

PDMS, a transparent, soft polymer, is widely used in chip manufacturing. Its elasticity allows for the integration of microvalves and micropumps, making it the preferred material for microfluidics. The relatively hydrophobic surface of PDMS, however, makes it easy to adsorb hydrophobic samples for testing or produce bubbles in the channel. Surface modification techniques are expected to address these issues.^57−59^ Compared to glass materials, microorganisms can grow on PDMS chips due to their breathability.^60^ Therefore, it is often used in research related to the culture, isolation, and detection of microorganisms. By integration of microvalves and microchannels on a chip, a high-throughput method of plant disease detection can be achieved. A PDMS chip using LAMP-related methods successfully detected DNA and RNA viruses from tomato and cucurbit plants, showing the potential for autonomous sample distribution and multiple gene diagnosis. PDMS-based microfluidic electrochemical devices designed by Freitas et al. have demonstrated ultrasensitive magnetic immunoassays for citrus tristeza virus with a detection limit of 0.3 fg mL^–1^ in the concentration range of (1.95–10.0) × 10^3^ fg mL^–1^.^61^ These works showed that the application of PDMS chips in early plant disease detection is promising.

### 4.2 Paper-Based Devices as a Lab on a Chip for Discovering Plant Diseases

As a cost-effective and portable material, paper can be used for manufacturing flexible devices. Due to their low cost, lightweight, and portability, lateral flow dipsticks (LFDs) once dominated the field of rapid detection, especially in modern medical monitoring, such as COVID-19 test strips. Microfluidic paper-based analytic devices (μPADs) are novel microfluidic devices that use filter paper as a carrier and utilize the hydrophilic nature of paper for capillary-driven fluid transport.^62^ The white background of the paper does not interfere with the discrimination of the results of the colorimetric reaction. Paper-based microfluidics are suitable for colorimetric detection, electrochemical detection, optical detection, and other analytical methods. In recent years, they have been widely used in the fields of food safety, environmental pollutant detection, and infectious disease detection. Wei et al. developed a micropaper-based gene sensor for the visual detection of banana bunchy top virus, which had a detection limit of up to 0.13 aM for gene fragments.^52^ This shows that the material chip is portable and fast, which means that it has the potential to successfully detect plant diseases.

### 4.3 Microfluidics Based on Hydrogels for Plant Disease Screening

Growing bacteria on an agar plate is the gold standard for bacterial analysis, but this method is laborious and time-consuming. Due to their oxygen permeability, most microfluidics with cell culture capabilities are manufactured using PDMS.^63^ Hydrogels are porous 3D materials with high water content that are formed by cross-linking hydrophilic polymers in water.^64^ Due to their biocompatibility and encapsulation, hydrogel structures can enhance substance transport and diffusion.^64^ Microfluidics made from hydrogels allows for simplified microbial culture and on-chip analysis.^63^ By providing nutrients, antibiotics, and indicators in the gel, sample handling steps can be eliminated and are microbiome specific. Moreover, oxygen can diffuse through the gel to the culture chamber, allowing for simple control of the oxygen conditions. In addition, because some types of hydrogels can respond to external environmental stimuli, hydrogel chips can be integrated with other technologies, such as optical analysis and nanotechnology, and thus have great potential in the culture and detection of microorganisms.

Aflatoxin B_1_-sensitive smart DNA hydrogels integrated with microfluidics demonstrated sensitive and user-friendly sensing devices. This approach combines gold nanoparticles and hydrogels for quantitative detection using a distance readout method.^65^ Although microfluidics made of hydrogel materials are rarely used in plant disease detection, this approach could lead to interesting findings.

### 4.4 Advanced Methods for Microfluidics Production

#### 4.4.1 3D Printing

3D printing enables the rapid prototyping of microfluidic devices, leading to time and cost savings. It allows for the creation of intricate structures, enhancing the manufacturing flexibility and precision. In particular, its layer-based manufacturing process allows for the design of geometric shapes, expanding the application of microfluidics.^66^ By leveraging 3D printing, diverse functionalities can be integrated into microfluidic devices, including sample processing, mixing, and separation, thereby enhancing the overall performance of microfluidic systems.^67^

#### 4.4.2 Laser

Laser processing offers high precision and resolution, making it suitable for diverse materials.^68^ For example, femtosecond laser processing can produce complex shapes on a microscopic scale in a variety of transparent materials. It allows for the creation of complex structures and shapes according to design specifications, with fast production times. Additionally, it helps avoid surface damage and roughness often associated with mechanical contact.^69^

#### 4.4.3 Nanoengineering

Nanoengineering technology enables the fabrication of microfluidic devices with nanoscale channels, facilitating the efficient separation of minute samples.^70^ Moreover, surface treatment can be utilized to create specific nanostructures on the channel surface or control structures within microfluidic devices. Additionally, nanomaterials can be incorporated to enhance the functionality and performance of microfluidic devices.^71^

## 5 Application of Microfluidics for the Detection of Plant Diseases

Pathogen characteristics are linked to plant diseases and guide the corresponding detection principles. Fungal diseases, characterized by mold and spots on leaves, involve the spread of spores. Image processing techniques help identify spores of airborne plant diseases. Viruses, which have simple structures, change the plant shape and color after infection. Bacteria-produced toxins and invasion induce leaf spots and damage. For these spore-independent pathogens, a combination of immunoassay and electrochemical response is currently the most commonly used analysis method. Table 2 shows the microfluidics that have been used to detect fungi, viruses, and bacteria in crops.

### 5.1 Fungus

Fungi constitute 70–80% of the pathogens that threaten food security by causing plant diseases. Reliable identification is crucial for pinpointing causes of similar symptoms and enabling symptomatic treatment.

**Figure 3:**
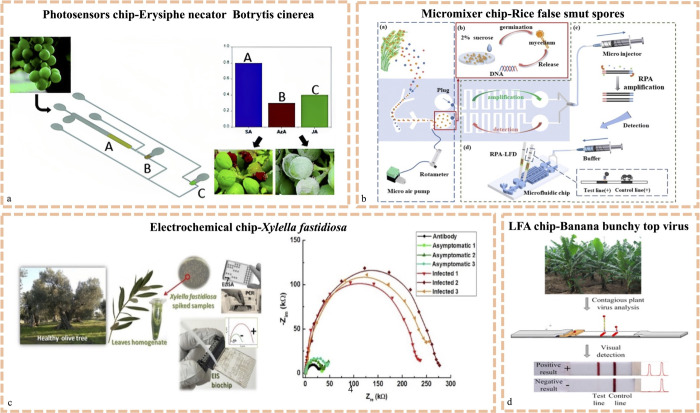
Detection target of the chip for detecting plant diseases. (a) Microfluidics for monitoring grape fungal infection by simultaneous detection of three plant hormones, SA, AzA, and JA. Reproduced with permission.^49^ Copyright 2020, Royal Society of Chemistry. (b) POCT test paper with the function of cultivating and monitoring the rice pathogen *Pseudomonas viridis*. Reproduced with permission.^50^ Copyright 2022, Royal Society of Chemistry. (c) LOC equipment to monitor infection with olive bacteria (*Xylella fastidiosa* subsp. *pauca* strain CoDiRO). Reproduced with permission.^51^ Copyright 2021, Multidisciplinary Digital Publishing Institute, sensors. (d) Lateral flow assay (LFA) device to identify the banana bunchy top virus. The materials were reproduced with permission.^52^ Copyright 2014, American Chemical Society.

In modern microfluidic fungus detection, plant stress analysis aids in discovering early biomarkers for disease occurrence. Biomarkers such as plant hormones and volatile organic compounds can be detected at an early stage of infection. Plants mainly rely on systemically acquired resistance for the immune response after pathogen infection. This process enhances organic acid production, inhibiting pathogen reproduction. Azelaic acid (AzA) and salicylic acid (SA) are common target metabolites for detecting plant infections. In a study by Eduardo et al., microbeads immobilized the enzyme tyrosinase in a microfluidic system with thin-film silicon photosensors, enabling real-time colorimetric detection of AzA inhibition of tyrosinase within a detection limit of 5–10 nm. This method revealed a 10^–3^-fold increase in the AzA concentration in infected grape samples compared to that in healthy ones.^72^ Jasmonic acid (JA) is considered another metabolic indicator by researchers.^73^ As shown in Figure 3a, simultaneous detection of three plant hormones, SA, AzA, and JA, was achieved using three recognition methods (nanoparticle conjugation, enzymatic reaction, and antibody–antigen recognition), and the detection limits within 7 min were 15, 10, and 4.4 μM, respectively.

Spectral detection and image processing technologies have provided promising methods for the accurate identification of known spores through statistical modeling. Different spores, varying in size, shape, and surface properties, exhibit distinct light absorption and reflection patterns during diffraction, resulting in characteristic streaks. The integration of a lens-free diffraction imaging system with microfluidics facilitates rapid detection of *Botrytis cinerea* spores in the air in a greenhouse with an average error of 6.42%.^74^ Furthermore, a microfluidic device that costs less than $150 and is constructed using standard soft-lithography technology can be used to effectively isolate and enrich disease-causing spores in rice.^75^ Using lens-free diffraction fingerprinting with a complementary metal-oxide semiconductor, this device produced quantifiable diffraction images of spores, with an average error rate of only 5.91%.

In the early stages of plant disease, detecting low concentrations of spores in the air is challenging. Therefore, it is necessary to pretreat the spores for separation and enrichment. Microfluidics using a composite field two-dimensional separation structure was used to enrich spores in greenhouse gas streams, with an enrichment efficiency of 88–94%.^76^ A straightforward active gas-driven microbial separation method utilizing focusing before separation can be used to effectively control particle movement.^77^ This method achieved a maximum clearance rate of 98% for fungal spores. Moreover, microfluidics with a double entrance and three-stage structure demonstrated the ability to diagnose rice fungal diseases by isolating and enriching *Magnaporthe grisea* spores and *Ustilaginoidea virens* spores in the air with 82.67% and 80.70% efficiency, respectively.^78^ A microfluidics chip comprising a half-wave pretreatment channel, inertial impactor, and low-pressure collection chamber facilitated separation based on spores of different sizes.^79^ Optimization of the design parameters resulted in sizes of 4.83 and 0.98 μm for this two-stage device.

As a fast technique for electrochemistry, impedance detection can also be combined with microfluidic systems to isolate fungal spores in the air. The average accuracy of a classification model based on four impedance characteristics was between 93.3 and 99.78%.^80^ Another study successfully used electrochemical impedance spectroscopy (EIS) to detect the pathogen *Sclerotinia sclerotiorum* in rapeseed.^81^

Due to its high specificity and sensitivity, nucleic acid detection is the main monitoring method for rice false smut spores (RFSS). However, the complexity and dependence on professional equipment prevent its implementation in portable devices due to the potential destruction of fungal spores. The microfluidic approach presented in Figure 3b involves the growth of the microbe on a chip and the use of the growing mycelium to detect *Ustilaginoidea virens*, achieving a sensitivity of 1 × 10^2^ to 1 × 10^5^ CFU mL^–1^.^50^

Nucleic acid detection methods are challenging when applied to fungal spores, because the spores have thick cell walls that are difficult to break and release nucleic acids. One solution to this problem is to culture the spores until they grow mycelium. In addition, most morphological examinations of fungal spores rely on advances in image recognition technology to achieve rapid and highly automated detection. However, the accuracy of this method is limited because the presence of particles of similar shape and size can easily lead to missed and false detection.

### 5.2 Virus

The monitoring of plant diseases caused by viral pathogens is highly important because of their potential to induce acute symptoms, quickly affect plant yield and quality, and result in economic losses. Viral infections are challenging to prevent, and it is difficult to avoid their impact on agricultural production after infection. The premise of this technology is accurate and rapid identification of infected crops and pathogen identification. Therefore, there is a critical need for fast, low-cost tools suitable for on-site crop virus monitoring that can benefit both botanists and farmers.

Figure 3d illustrates a compact flow-sensing biosensor for the rapid identification of banana bunchy top viruses. This paper-based sensor integrates chromatography and traditional immunoassay, ensuring a detection limit of 0.13 aM, which is 10 times greater than that of electrophoresis.^52^ For *Citrus tristeza* virus (CTV), a cost-effective and straightforward method involving rapid prototyping to fix antibodies on an electrode surface, enabling ultrasensitive electrochemical detection, is crucial for managing citrus production (detection limit: 0.3 fg mL^–1^).^61^

Currently, there are few portable methods that combine the collection and detection of viruses, and most of these assays require off-chip preprocessing. In addition, detection methods often require additional visualization steps, creating obstacles to the implementation of the POCT.

### 5.3 Bacteria

A microfluidic immune sensor with integrated electrochemistry can detect *Xanthomonas arboricola* (XA) in walnuts.^82^ Another electrochemical immunosensor for the efficient detection of *Pectobacterium atrosepticum* (Pba) in potatoes is based on impedance theory.^83^ This LOC platform combining microfluidic modules and microelectrode arrays had a detection limit as low as 10^4^ CFU mL^–1^ and a cost as low as 5 €, which means that it is more sensitive than traditional ELISA methods and less costly than PCR methods. Figure 3c illustrates a similar LOC microelectrode with specific antibodies, reporting 7.5 times greater sensitivity than ELISA for detecting the pathogenic bacterium that causes Olive Quick Decline Syndrome.^84^

In conclusion, the integration of POCT microfluidic technology with direct or indirect detection techniques is valuable for managing various crops. Recent efforts have focused on reducing the detection time, enhancing the sensitivity, and minimizing the sample volume. Modifying microchannels in sample enrichment areas remains a common strategy for improving the analyte enrichment efficiency. The optimization of detection methods is crucial for achieving excellent performance in field pathogen detection. In addition, the development of chips that integrate separation, detection, and visualization remains difficult.

## 6 Combining Artificial Intelligence (AI) and the Internet of Things (IoT) with LOC Devices—Innovation and Consummation

**Figure 4:**
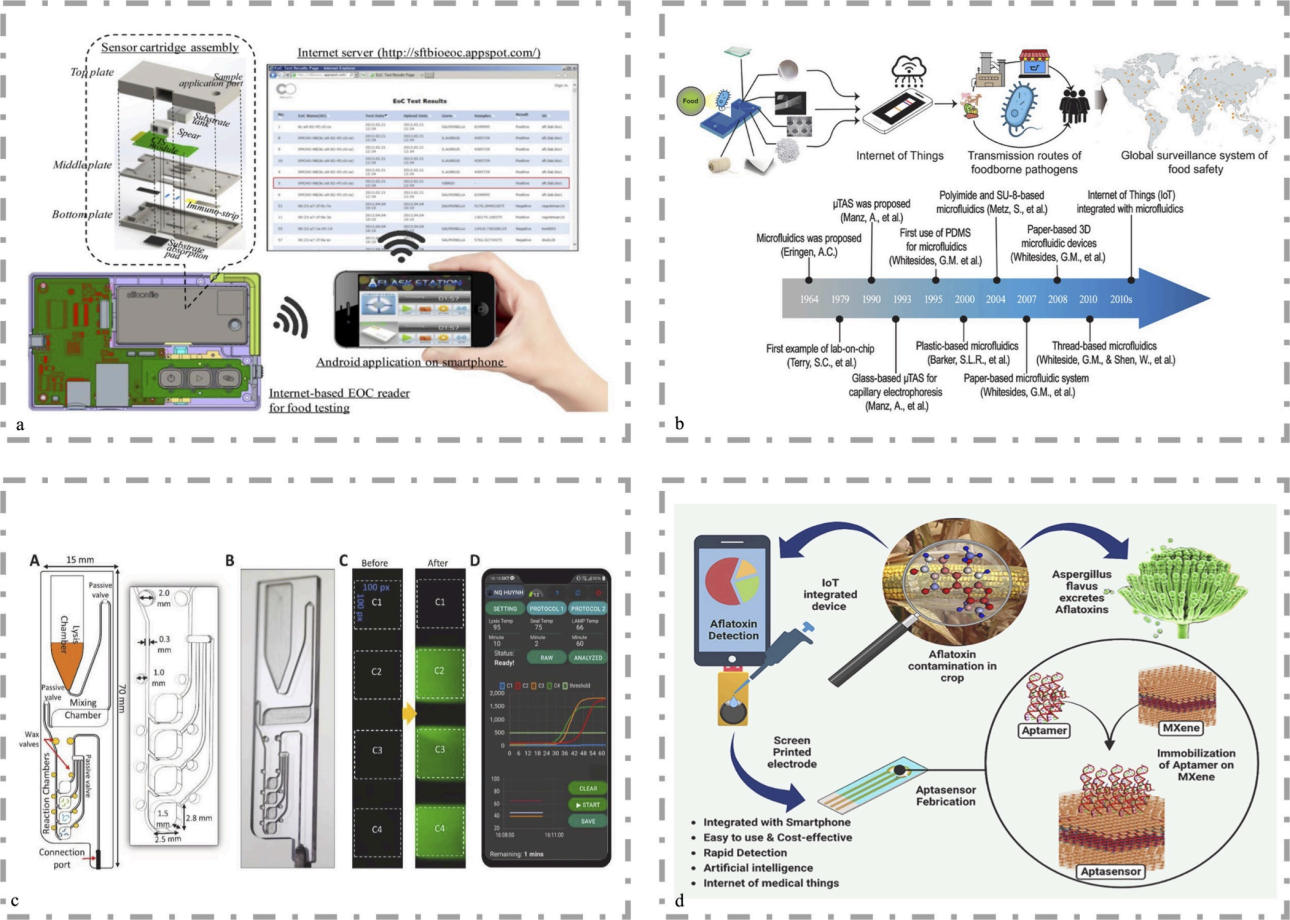
Integration of the IoT and AI with microfluidic devices. (a) CMOS image sensors (CIS) are used to detect signals generated by the immune analysis system to address detection issues hindering Internet of Things (IoT) monitoring. Reproduced from ref (85) with permission. Copyright 2016, Elsevier. (b) Advancements in microfluidic systems based on various materials (glass/silicon, thermoplastic plastics, hydrogels, paper, wires, combinations of various materials) in the detection of foodborne pathogens and their expansion in the era of “big data”. Reproduced from ref (54) with permission. Copyright 2023, Elsevier. (c) The Internet of Things (IoT) serves as an ideal platform for real-time screening of COVID-19 through point-of-care (POC) and ubiquitous healthcare monitoring for patients. Reproduced from ref (86) with permission. Copyright 2022, Elsevier. (d) Using ligand-based MXene biosensing technology coupled with artificial intelligence and machine learning for the design of ligand sensors and advanced data analysis methods for the detection of fungal toxins. Reproduced from ref (87) with permission. Copyright 2023, Elsevier.

The IoT facilitates intelligent data collection, processing, and response across various domains.^93^Figure 4a illustrates the utilization of a complementary metal–oxide–semiconductor (CMOS) image sensor to detect signals generated by an immunological analysis system, thereby addressing the detection issues hindering loT monitoring. Figure 4b shows the advancement of microfluidic systems based on various substrates. Figure 4c presents an innovative IoT-based POCT device for real-time screening and continuous monitoring in healthcare. Figure 4d demonstrates the integration of IoT with biological sensing devices. Smartphone-enabled colorimetric tests, such as detecting *Botrytis cinerea* and *Erysiphe necator* in grapes, have shown IoT’s effectiveness in plant disease detection.^94^

The integration of microfluidics with artificial intelligence and the Internet of Things offers a breakthrough approach for detecting plant diseases, providing numerous advantages and transformative potential for agricultural management. By combining microfluidic devices with AI algorithms and IoT sensors, researchers can develop advanced diagnostic systems capable of the rapid, accurate, and automated detection of plant pathogens. This integration helps achieve real-time monitoring of crop health, enabling early detection and proactive disease management strategies. The synergy of microfluidics with AI, particularly deep learning techniques such as convolutional neural networks (CNNs), has led to significant progress in image processing for plant disease identification. Transfer learning, as demonstrated in cassava disease detection, is a cost-effective and deployable technology.^95^ Another study combined a deep neural network (DNN)-based model with the circle Hough transform for accurate fluorescent droplet measurement via digital polymerase chain reaction (dPCR) analysis.^96,97^

Furthermore, the integration of microfluidics with AI and the IoT has enabled the development of smart farming systems that optimize resource utilization and improve crop yield and quality.^98^ AI algorithms can analyze data collected from microfluidic sensors and IoT devices to provide insights into environmental conditions, plant physiology, and disease dynamics, allowing farmers to make data-driven decisions regarding irrigation, fertilization, and disease control.^99^ Additionally, the remote accessibility afforded by IoT-enabled microfluidic devices enables farmers to monitor multiple crops across large agricultural fields, enhancing the efficiency and scalability of disease detection and management efforts.

However, despite these advantages, there are several limitations and challenges associated with the integration of microfluidics, AI, and the IoT for plant disease detection.^100^ One significant challenge is the need for reliable microfluidic devices capable of performing sensitive and specific detection of plant pathogens in complex environmental samples. Additionally, the development of AI algorithms for disease diagnosis requires extensive training data and validation to ensure accuracy and reliability across diverse plant species. Moreover, the deployment of IoT-enabled microfluidic systems in agricultural settings may face challenges related to connectivity, power supply, and data security, particularly in remote or resource-limited areas.

In summary, the coupling of microfluidics with AI and IoT represents a promising approach for revolutionary plant disease detection and agricultural management. Despite the challenges and limitations to overcome, the potential benefits in terms of early detection, precision management, and sustainable agriculture make this integration a compelling area for future research and development in the field of agriculture.

## 7 Discussion and Perspectives

As mentioned above, LOC-based detection systems are promising for the early detection of various pathogens. Sample preparation is a crucial domain in which microfluidic devices are able to make significant contributions to future portable technologies. In the context of plant samples, this procedural step assumes paramount importance, as it serves to augment the sample’s richness, eliminate inhibitory elements, and concentrate pathogens. Another utilization of multilayer structures in microfluidic platforms that can be effectively employed is sample preparation and filtration, which involves the integration of micropillars.^101^ According to different analysis objectives, the analysis cost can be reduced by improving the separation strategy. In addition, higher sensitivity than traditional methods can also be achieved because of the combination of different detection methods. High-throughput platforms were deployed through the integration of parallel microchannels, micropumps, and microvalves, while also harnessing diverse methodologies for cell/molecule entrapment and transport. Therefore, the application of microfluidic technology will be conducive to improving the agricultural production efficiency and food safety.

The combination of microfluidics with advanced molecular, serological, and imaging techniques provides a broad scope for new ideas in plant pathology detection. However, the realization of its wide application still faces some problems. At present, most microfluidic systems are able to achieve a single task with some degree of success, but the integration of various functions such as separation, detection, and output is currently a major technical challenge. In addition, the requirements of droplet manipulation for multiplexing and automation are often not proper. While combining CRISPR with microfluidic systems can enhance multiplexing, deploying them widely in a short time remains difficult. The commercialization of parallelized sensor and array devices needs to be balanced with portability. As one of the most commonly used chip materials, the hydrophobicity of PDMS enables the aggregation and blockage of hydrophobic molecules. In addition, their structures may be changed in some organic solvents. In addition, with the emergence of an increasing number of microfluidics with different functions, the sensitive and reliable detection of extremely low pathogen levels still needs further exploration.

Despite the optimistic prospects, there are several areas of optimization that could enhance the future potential of microfluidics in the agri-food sector. (i) Currently, research objects for microfluidic disease detection of agricultural products are limited, as shown in Table 2. However, consideration should be given to exploring a wider variety of agricultural products that are of economic value to humanity. Additionally, since the same plant can be threatened by multiple pathogens, microflow chips that can detect multiple diseases at the same time should be developed. (ii) The existing lab-on-a-chip requires optimization in terms of power consumption, detection speed, and cost to meet the needs of POCT for real-time detection. Future research on biochemical detection methods should prioritize quantitative and high-throughput detection, which is crucial for early disease discovery. The integration of pathogen isolation, detection, and results visualization can enhance the portability of plant disease detection for field workers. Moreover, the development of CRISPR/Cas for POCT of plant RNA viruses shows promise.^102^ Additionally, new challenges associated with microfluidics include potential cross-reactivity and the need for appropriately designed amplification channel volumes. To address some of these challenges, future work can focus on developing fully automated microfluidic assays through integrated processes. (iii) There is great promise in the development of novel microfluidic techniques for effectively separating the DNA content of plant diseases from their host origins. The presence of contamination with sequences of host origin not only limits the sensitivity of portable devices but also complicates bioinformatics analysis for pathogen detection.^103^ (iv) The use of new materials may also increase the sensitivity of such devices. The research and application of microfluidics are still in the initial stage, and it is expected to become an opportunity to improve the quality of agricultural food. With the advent of Agriculture 4.0, the development of this field will contribute greatly to world food production.

## Data Availability

Data will be made available upon request.
